# Effect of foot load changes on foot arch evaluation using foot pressure distribution data

**DOI:** 10.1186/1757-1146-7-S1-A114

**Published:** 2014-04-08

**Authors:** Kazuya Imaizumi, Yumi Iwakami, Kazuhiko Yamashita

**Affiliations:** 1 Division of Healthcare Informatics, Faculty of Healthcare, Tokyo Healthcare University, Tokyo, 154-8568, Japan

## Background

The foot arch serves important functions in regard to shock absorption and the action of walking. Simple and quantitative classification of foot arch types such as flat foot and high arch would be helpful in health support for the elderly. The present authors have developed a classification system for foot arch type showing high reliability using foot pressure distribution data [1,2]. However, effect of foot load changes on foot arch evaluation remains unclear. The aim of this study was to investigate the effect of foot load changes on foot arch evaluation using foot pressure distribution data.

## Method

We conducted a field test involving elderly individuals. Foot pressure distribution data were obtained by the field test with elderly subjects standing on 1 leg and 2 legs. A total of 44 healthy elderly Japanese subjects (2 males, 42 females) attended sessions on foot care in Tokyo. Their mean age was 70.7 ± 7.0 years, mean height was 153.1 ± 5.5 cm, and mean weight was 49.9 ± 4.3 kg. Subjects were first requested to stand on both legs with eyes open. When subjects were adjudged stable in the standing position, digital foot pressure distribution data were obtained by using MAT-SCAN (Nitta Corporation, Japan).

Subjects were next requested to stand on their right leg only with eyes open, and data were obtained as above. Based on our previous study [2], mfp under both bilateral and unilateral stance was calculated. Subjects were categorized into 3 groups according to data from bilateral stance: high arch, normal arch, and flat foot. Paired t-test for mfp between the 2 stances was implemented for all 3 groups. The level of significance of the test was set at 5%.

## Results and discussion

**Figure 1:**
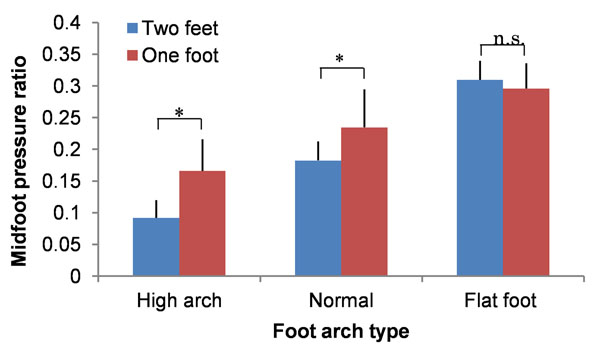
Midfoot pressure ratio (mfp) in the 3 arch groups for both bilateral and unilateral stance

Among 44 subjects, 10 were categorized as having high arch and 8 as having flat foot according to bilateral stance data. Figure 1 shows mfp for both stances. Paired t-test showed a significantly higher mfp for unilateral than bilateral stance in the high and normal arch groups (p = 0.002 and p <0.001, respectively). In the flat foot group, no significant inter-stance difference was seen. Thus, in the normal and high arch groups, it is assumed that the foot arch was deformed by increased foot arch load in unilateral stance, thus altering mfp. On the other hand, in the flat foot group, it is assumed that foot arch structure was reduced and foot function consequently weakened.
